# Secret of Eloquence

**Published:** 1873-11

**Authors:** 


**Secret of Eloquence.**—I owe my success in life to one single fact, namely: At the age of twenty-seven I commenced, and continued for years, the process of daily reading and speaking upon the contents of some historical or scientific book. These off-hand efforts were made sometimes in a corn-field, at others in the forest, and not unfrequently in some distant barn, with the horse and cow for my auditors. It is to this early practice in the great art of all arts that I am indebted for the primary and leading impulses that stimulated me forward, and shaped and molded my entire subsequent destiny. Improve, then, young gentlemen, the superior advantages you here enjoy. Let not a day pass without exercising your powers of speech. There is no power like that of oratory. Cæsar controlled men by exciting their fears; Cicero, by captivating their affections, and swaying their passions. The influence of the one perished with its author; that of the other continues to this day.—Henry Clay.

